# Enhanced immunogenicity of leukemia-derived exosomes via transfection with lentiviral vectors encoding costimulatory molecules

**DOI:** 10.1007/s13402-020-00535-3

**Published:** 2020-06-23

**Authors:** Weiwei Hu, Fang Huang, Liuxin Ning, Jun Hao, Jiangbo Wan, Siguo Hao

**Affiliations:** 1 Department of Hematology, Xinhua Hospital Affiliated to Shanghai Jiaotong University School of Medicine, 1665# Kongjiang Road, Shanghai, 200090 China; 2 Interdisciplinary Oncology Program, Faculty of Medicine, University of British Columbia, Vancouver, BC Canada; 3 Department of Experimental Therapeutics, BC Cancer Agency, Vancouver, BC Canada

**Keywords:** Leukemia, Exosomes, Costimulatory molecules, Immunotherapy

## Abstract

**Background**: Tumor cell-derived exosomes (TEXs) have been widely used to induce antitumor immune responses in animal models and clinical trials. Similarly, leukemia cell-derived exosomes (LEXs) can induce antileukemia immune responses in animal models. However, the antileukemia immunity induced by LEXs is less effective, which may be due to an inadequate costimulatory capacity.

**Methods**: In this study, we transduced L1210 leukemia cells with a lentiviral vector encoding two B7 costimulatory molecules (CD80, CD86) and obtained LEXs that highly expressed CD80 and CD86. The antileukemia immune response derived from these LEXs was examined in vitro and in vivo in animal models.

**Results**: We found that B7 gene-modified LEXs, including LEX-CD80, LEX-CD86, and LEX-8086, could significantly boost the expression of CD80 and CD86 in dendritic cells (DCs) and promote the secretion of functional cytokines such as TNF-α and IL-12. Moreover, these B7 gene-modified LEXs, particularly LEX-CD8086, could effectively induce CD4^+^ T cell proliferation, Th1 cytokine secretion, and an antigen-specific anti-leukemia cytotoxic T lymphocyte (CTL) response. Additional animal studies indicated that immunization with B7 gene-modified LEXs, in particular LEX-CD8086, could significantly retard tumor growth compared to the control LEXnull group.

**Conclusions:** This study sheds light on the feasibility of obtaining LEXs that overexpress costimulatory molecules via genetically modified leukemia cells, thereby enhancing their anti-leukemia immunity and providing a potential therapeutic strategy that contributes to leukemia immunotherapy.

## Introduction

Recently, remarkable progresses have been made in the clinical treatment of leukemia, and these are mainly attributed to high doses of chemotherapy and hematopoietic stem cell transplantation [1, 2]. However, several patients, especially the elderly, still suffer and cannot benefit from these strategies [3]. Therefore, novel and tolerable treatments for leukemia are urgently needed. Immunotherapy has revolutionized leukemia therapy in the past few years by, for instance, using chimeric antigen receptor T cell (CAR-T) therapy [4] or immune checkpoint inhibitors, which are two successful therapeutic strategies in this field [5–9]. Additionally, the use of anti-tumor vaccines represents another remarkable type of immune therapy which inhibits leukemia relapse and promotes leukemia-free survival [10, 11].

Previously we found, along with others, that most tumor cells secrete exosomes and that tumor cell-derived exosomes (TEXs) are highly enriched in tumor-associated antigens as well as in a series of immune-related proteins such as histocompatibility complex molecules and heat shock proteins [12–16]. Therefore, TEXs are considered as promising antitumor vaccines. Like tumor cells, leukemia cells also release exosomes. These leukemia cell-derived exosomes (LEXs) may harbor leukemia cell-associated antigens that can induce anti-leukemia immunity in vitro and in vivo [17, 18]. However, the anti-leukemia immunity induced by LEXs is still less effective, which may mainly result from an inadequate costimulatory capacity [19]. Therefore, LEX-based anti-leukemia vaccines still need to be optimized in order to improve their immunogenicity. Some studies have used vectors encoding B7–1 (CD80), ICAM-1, and LFA-3 to efficiently overexpress these costimulatory molecules in dendritic cells (DCs) and leukemia cells, after which an increased antigen-presenting potency of these cells was observed compared to their uninfected counterparts, as demonstrated by enhanced T cell responses [20, 21]. Another study showed that enhanced immunogenicity targeting chronic lymphocytic leukemia (CLL) cells could be obtained via transfection with vectors encoding multiple costimulatory molecules [22]. Based on these observations, we hypothesized that a high expression of costimulatory molecules on LEXs may enhance the anti-leukemia immunity of LEX-based vaccines.

B7 proteins, including B7–1 (CD80) and B7–2 (CD86), are classical costimulatory molecules that are expressed on the surface of antigen-presenting cells (APCs). They can interact with the co-receptor CD28, which is constitutively expressed on the surface of both naive and activated CD4^+^ T cells. It has been found that cytotoxic T cells better eradicated murine malignancies when transfected to express CD80 and CD86, thus demonstrating a role of B7 proteins in antitumor immunity [23]. A previous report also demonstrated that antigen presentation by malignant blood cells for the activation of cytotoxic T cells could be strengthened through CD80 cDNA transfection [24], whereas others modified DCs by RNA interference targeting CD80 and CD86 leading to a low T cell responsiveness via enhanced T cell apoptosis [25].

In this study, CD80 and CD86 were overexpressed in L1210 leukemia cells through transduction of lentiviruses encoding CD80 and CD86. LEXs were purified from the supernatants of the transfected cells. We found that transduced leukemia cell-derived LEXs (LEX-CD80, LEX-CD86, LEX-CD8086) carried ectopic levels of CD80 and CD86. These B7 gene-modified LEXs could efficiently promote CD4^+^ T cell proliferation, stimulate Type I cytokine secretion, and induce an antigen-specific anti-leukemia cytotoxic T lymphocyte (CTL) response, leading to a stronger anti-leukemia protective immunity in vivo.

## Materials and methods

### Reagents

RPMI-1640 medium, fetal bovine serum (FBS), and serum-free medium were all purchased from Invitrogen (Shanghai, China). Rabbit anti-mouse hsp70, CD9, CD63, and CD81 antibodies were purchased from Cell Signaling Technology (Shanghai, China). Recombinant mouse granulocyte-macrophage colony-stimulating factor (rmGM-CSF), recombinant mouse interleukin rmIL-4, rmIL-2, and lipopolysaccharide (LPS) were purchased from PeproTech (Shanghai, China). PE-labeled anti-CD11c, PE-cyanine7 conjugated anti-CD80, and APC-labeled anti-CD86 were purchased from eBioscience (Shanghai, China). EasySep™ Mouse CD4^+^ and CD8^+^ T cell isolation kits were purchased from Stem Cell Technologies (Vancouver, Canada).

### Cell lines and animals

L1210, an acute lymphoblastic leukemia cell line from DBA/2 mice, was purchased from the Chinese Academy of Sciences (Shanghai, China). In order to avoid the influence of FBS exosomes, L1210 cells were cultured in RPMI-1640 medium containing 10% exosome-free FBS, 100 μg/ml penicillin, and 100 mg/ml streptomycin at 37 °C in a sterile incubator containing 5% CO_2_. Six to eight-week-old DBA/2 female mice were purchased from the Shanghai SLAC Laboratory Animal Center (Shanghai, China) and kept under standard specific-pathogen-free (SPF) conditions. All animal experiments were conducted according to the guidelines of the Ethics Committee of Xinhua Hospital, affiliated to the Shanghai Jiao Tong University School of Medicine, Shanghai, China.

### Lentivirus vector construction

Two self-complementary oligonucleotide pairs carrying sequences that target mouse CD80 and CD86 were designed and synthesized (Shanghai Hanbio Co., Ltd., Shanghai, China). The CD80 and CD86 paired oligonucleotides were separately ligated into lentiviral frame plasmids. The recombinant plasmids were transformed into *Escherichia coli* cells and positive clones were selected by PCR. 293T cells were co-transfected with the recombinant lentiviral vectors (1.7 μg), pSPAX2 vector (1.13 μg), and pMD2G vector (0.57 μg) in order to produce lentivirus particles. 72 h after transfection, supernatants containing lentiviral particles were harvested, filtered through a 0.45-μm membrane, and concentrated via ultracentrifugation. The lentiviral vectors expressed the green fluorescence and anti-puromycin proteins. Fluorescence intensity and anti-puromycin screening tests were used to ensure virus titration and lentiviral infection efficiency measurements. CD80 and CD86 interference efficiencies were assayed by RT-qPCR and flow cytometry. The CD80 sequences that were used were as follows: forward strand: AGCTGTGACCGGCGCCTACATGGCTTGCAATTGTCAGTTG; reverse strand: TTCCTCTGCCCTCAGCGGCCAAGGAAG ACGGTCTGTTCAGC. The CD86 sequences were as follows: forward strand: ATGGACCCCAGATGCACCAT; reverse strand: TCACTCTGCATTTGGTTTTG.

### Transduction of leukemia cells and isolation of exosomes

L1210 cells were pre-cultured in complete medium containing 10% exosome-free FBS for 24 h in order to avoid contamination from the serum. L1210 cells were transduced with recombinant lentiviral vectors encoding CD80 and CD86 for 72 h, after which the culture supernatants were collected for the isolation of exosomes as reported previously [26]. Exosomes that were derived from non-manipulated L1210 cells were named control LEX, while exosomes that were purified from the supernatants of L1210 cells transduced with the lentiviral vectors carrying CD80, CD86 or both were named LEX-CD80_,_ LEX-CD86_,_ and LEX-CD8086, respectively. LEXs purified from the supernatant of L1210 cells transduced with a null vector were named LEXnull.

### Flow cytometry

To quantify the expression of CD80 and CD86 in LEXs, 30 μg of LEX preparations were incubated with aldehyde/sulfate latex beads (Invitrogen, Shanghai, China) at 4 °C overnight. Next, 100 mmol/L glycine was added to block the reaction. The exosome-loaded beads were washed twice in PBS buffer containing 1% FBS and then stained with specific antibodies directed against mouse CD80 and CD86 or the isotype control. Subsequently, the prepared samples were analyzed using a FACScan. In order to analyze DC phenotypes, the cells were concentrated, washed with PBS twice, and next incubated with fluorescent-conjugated monoclonal antibodies (mAb) directed against CD80, CD86, and CD11c for 30 min at room temperature.

### Electron microscopic and Western blot analyses of exosomes

Exosome samples were stained according to negative staining procedures and the morphology of exosome preparations was visualized using a Philips CM12 transmission electron microscope operating at 80 kV. Western blot analysis of the typical exosome markers CD9, CD63, and Hsp70 was conducted as previously described [27]. The primary antibodies used were all purchased from Cell Signaling Technology (Shanghai, China). Exosomes were enriched at high concentrations for an efficient protein extraction and the results were analyzed using Image Lab software.

### Cytokine assay

Tumor necrosis factor-α (ΤΝF-α) and IL-12 levels in DC supernatants (48 h after coculture with LEXs), as well as IFN-γ, ΤΝF-α and IL-2 levels in CD4^+^ T cell supernatants (48 h after stimulation) were detected using ELISA kits according to the manufacturer’s instructions (Westang, Shanghai, China).

### CD4^+^ T cell proliferation assay

Splenic CD4^+^ T cells of mice immunized with LEX-CD80_,_ LEX-CD86_,_ and LEX-CD8086 were isolated 7 days after the last immunization using an EasySep™ mouse CD4^+^ T cell isolation kit according to the manufacturer’s instructions (Stem-cell Technologies, Vancouver, Canada). Next, the purified CD4^+^ T cells (1 × 10^5^/well) were co-cultured with irradiated L1210 cells (1 × 10^4^/well) or with p388 cells (1 × 10^4^/well) as a control target for 72 h with 20 μg/ml phytohemagglutinin (PHA) at 37 °C and 5% CO_2_. [^3^H] thymidine (0.5 μCi/well) was then added to the medium after which incubation was carried out for 16 h. Subsequently, the cells were harvested and ^3^H-thymidine incorporation was measured by liquid scintillation spectroscopy using a MicroBeta counter (Beckman Coulter, Krefeld, Germany). The results are reported as mean counts per minute (cpm) and the standard error of the mean (SEM). All assays were conducted in triplicate.

### Cytotoxicity assay

Cytotoxic responses were evaluated according to the manufacturer’s instructions (Promega, Madison, WI, USA). Briefly, splenic CD8^+^ T cells were isolated from mice immunized with PBS or with 10 μg LEX-CD80, LEX-CD86, LEX-CD8086 or LEXnull. Seven days after the last stimulation, splenic CD8^+^ T cells were isolated from immunized mice using an EasySep™ mouse CD8^+^ T cell Isolation Kit (Stem-cell Technologies, Vancouver, Canada). Cells were re-stimulated with irradiated L1210 cells for 7 days and then harvested as effector cells. L1210 cells were used as specific target cells and p388 cells were used as controls. The cells were seeded in a 96-well plate at 1 × 10^4^ cells/well. After adding CytoTox 96^®^ Reagent (Promega, Madison, WI, USA), absorbance at 490 nm was recorded and cells were incubated for 30 min, protected from light. The magnitude of the cytotoxic response at different effector/target (E/T) ratios was evaluated using a LDH assay (Promega, Madison, WI, USA), and specific lysis (%) was calculated as follows: (experimental LDH release − effector cells − target spontaneous LDH release)/(target maximum LDH release) × 100 [28].

### Animal studies

To evaluate the protective immunity of costimulatory molecule gene-modified LEXs, PBS or 10 μg LEX-CD80_,_ LEX-CD86, LEX-CD8086 or LEXnull were subcutaneously (s.c.) injected at the inner side of the right hind thighs of DBA/2 mice on day 0. The immunization was boosted twice, on day 7 and day 14. On day 21, the immunized mice were s.c. challenged with L1210 cells (0.5 × 10^6^ cells/mouse) at the outer side of the same lateral thighs. Survival rates and tumor sizes (calculated as length × width^2^ xπ/6) were assessed every 2 days [28].

### Generation of bone marrow-derived DCs and coculture with LEXs

To investigate the effect of B7 gene-modified LEXs on the biologic properties of DCs, we examined the immunophenotype and the secretion of cytokines by DCs cocultured with LEXs. The generation of bone marrow-derived DCs from DBA/2 mice has been reported before [29]. Following cell culture for 6 days, DCs were collected and cocultured with the above mentioned LEXs in complete medium containing 10% exosome-free FBS for 24 h. DCs cultured with LPS or without stimulation were used as controls. DCs and supernatants were collected and stored.

### Statistical analysis

Experiments were performed at least three times. Data are presented as the mean ± SD or SEM. The log-rank test was used to analyze survival data and differences between the two groups were analyzed with Student’s t test. Statistical significance was determined as *p* < 0.05.

## Results

### LEXs highly expressing CD80 and CD86 can be obtained from B7 gene-modified L1210 leukemia cells

**Fig. 1:**
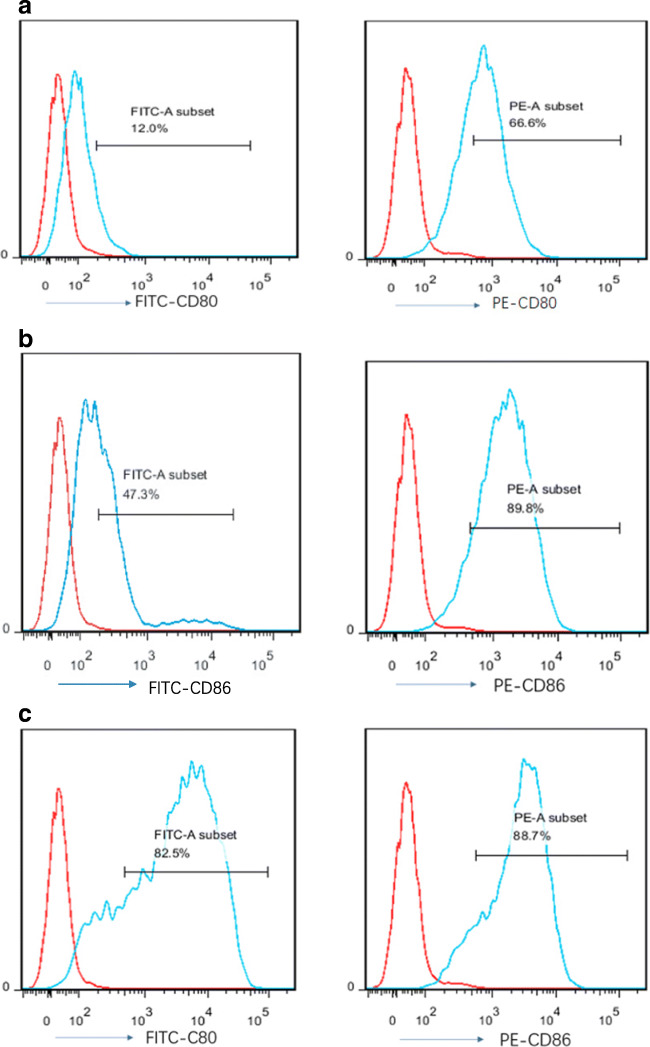
Immunophenotype analysis of B7 gene modified-L1210 cells. (**a**) Unmodified L1210 cells. (**b**) L1210 cells transfected with lentiviral vectors carrying CD80 or CD86 genes. (**c**) L1210 cells transfected with lentiviral vectors carrying both CD80 and CD86 genes. Data are representative of three independent experiments

**Fig. 2:**
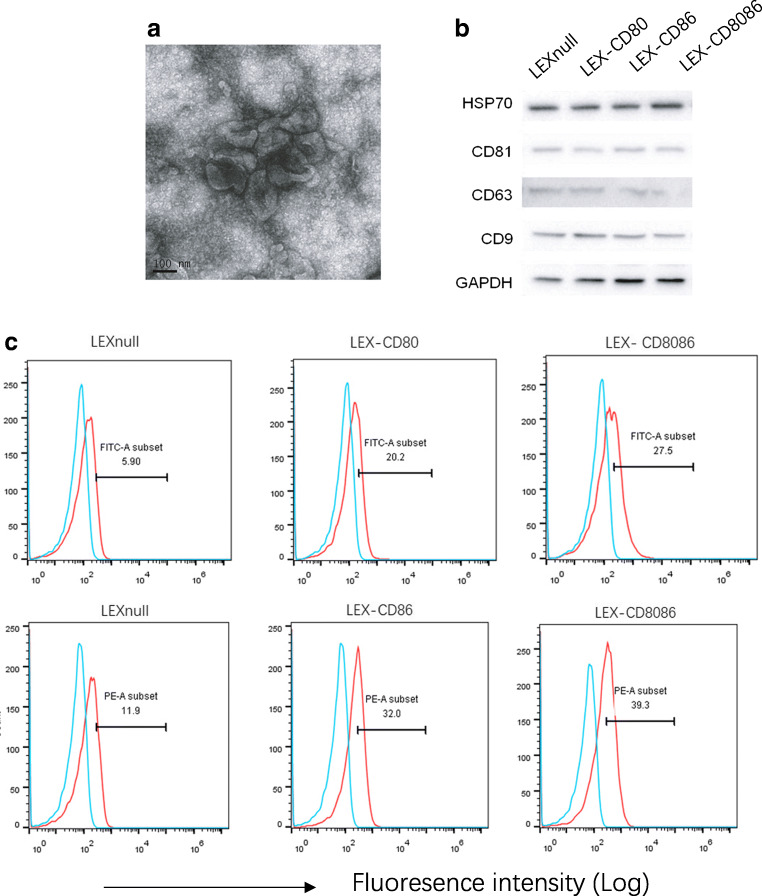
Characterization of LEXs derived from B7 gene modified-L1210 cells. (**a**) Exosomes derived from B7 gene modified-L1210 cells visualized using electron microscopy. (**b**) Western blot analysis of typical exosomal proteins. (**c**) Expression of CD80 and CD86 on B7 gene modified-LEXs measured by flow cytometry. The experiments were performed in triplicate. One representative experiment is shown

To confirm whether LEXs highly expressing CD80 and CD86 could be obtained from the B7 gene-modified leukemia cells, L1210 leukemia cells were transduced with lentiviral vectors carrying either B7–1 (CD80) or B7–2 (CD86), or the two genes. As shown in Fig. 1, after transduction, CD80 and CD86 expression in L1210 cells was markedly increased (Fig. 1a, b), particularly when cells were transduced with the two genes (Fig. 1c). The levels of CD80 and CD86 in LEXs derived from transduced cells were detected by flow cytometry to examine whether LEXs derived from B7 gene-transduced cells also express high levels of CD80 and CD86. As shown in Fig. 2, the expression of CD80 in LEX-CD80 and of CD86 in LEX-CD86 were significantly higher than those in LEXnull, indicating that LEXs derived from CD80- and CD86-overexpressing cells also overexpress CD80 and CD86. To examine whether gene transduction would affect the biology of LEXs, we examined LEXs derived from modified L1210 cells regarding morphology and the expression of related proteins using electron microscopy and Western blot analysis. As shown in Fig. 2a and b, LEXs exhibited diameters ranging from 40 to 100 nm and a plate-shaped characteristic morphology. Western blot analysis indicated that all the LEXs derived from B7 gene-modified L1210 cells fully expressed Hsp70, CD9, CD63, and CD81, which are considered typical exosomal proteins. Meanwhile, the expression of CD80 and CD86 on the modified LEXs was confirmed by flow cytometry (Fig. 2c). Together, these data suggest that B7 gene transfection did not affect the biological properties of exosomes derived from transfected leukemia cells.

### B7 gene-modified LEXs can promote CD4^+^ T cell proliferation and induce a potent antigen-specific anti-leukemic CTL response

**Fig. 3:**
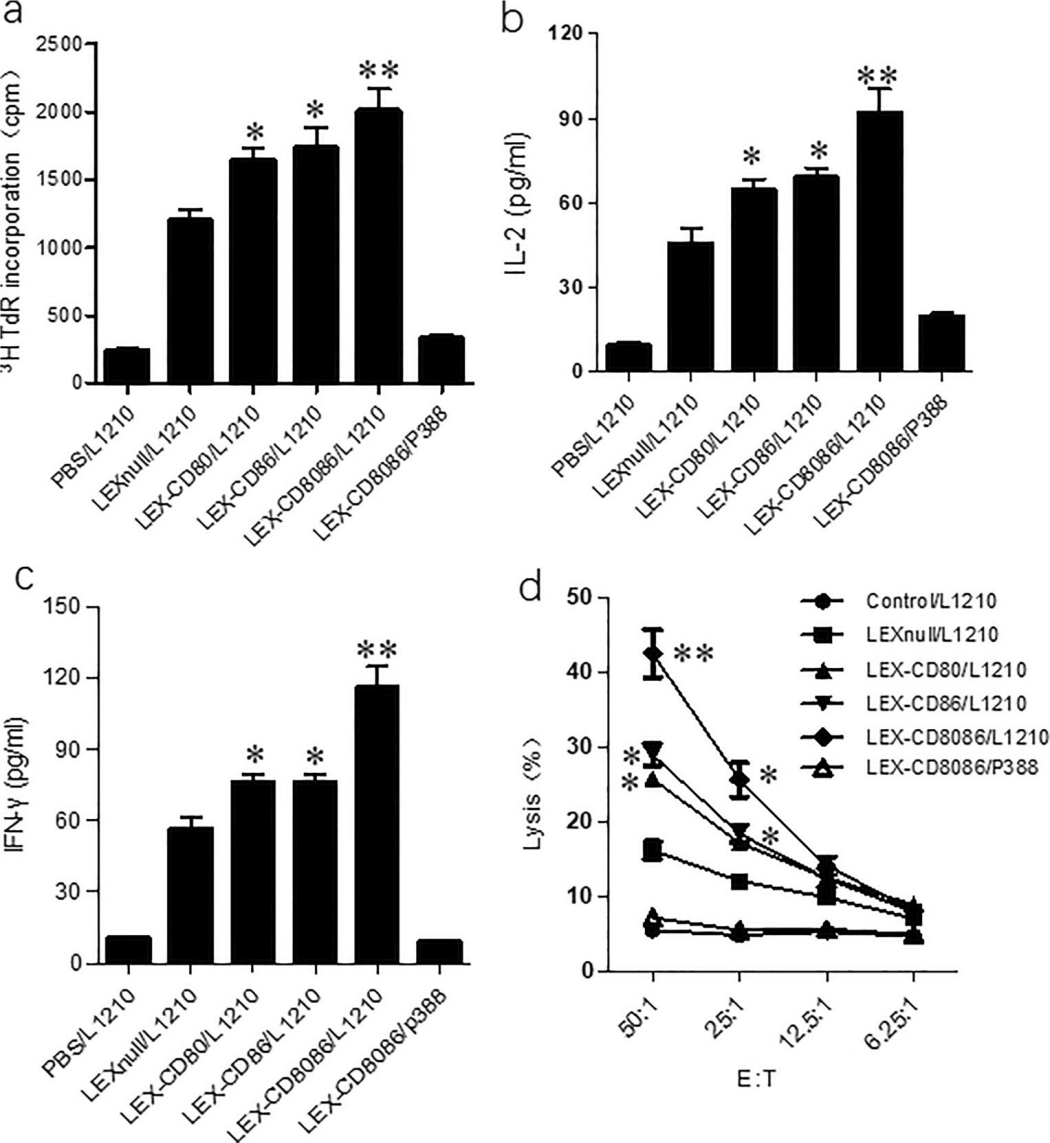
CD4^+^ T cell proliferation and cytotoxicity assays. Splenic CD4^+^ T cells were isolated from immunized DBA/2 mice incubated with irradiated L1210 cells or p388 cells. T cell proliferation was evaluated by ^3^H thymidine incorporation (**a**). Secretion of IL-2 (**b**) and IFN-γ (**c**) in culture supernatants was determined by ELISA. CD8^+^ T cells were isolated from immunized DBA/2 mice and referred as effectors. L1210 cells served as target cells and cytotoxicity assays were performed at different effector/target cell ratios (**d**). *p < 0.05 and **p < 0.01 denote statistically significant differences. Data are representative of three independent experiments

CD4^+^ T cells play a pivotal role in antitumor immune responses. To investigate the antigen-specific CD4^+^ T cell response induced by modified LEXs, splenic CD4^+^ T cells from mice immunized with LEX-CD80_,_ LEX-CD86, LEX-CD8086 and LEXnull were isolated and evaluated for their response to L1210 or p388 cells in vitro. We found that immunization with LEX-CD80, LEX-CD86, LEX-CD8086 and LEXnull induced a certain CD4^+^ T cell proliferation response to L1210 cells and not any response to p388 cells (Fig. 3a). Among them, the immunization with LEX-CD80_,_ LEX-CD86 and LEX-CD8086 more significantly boosted CD4^+^ T cell proliferation compared to that with LEXnull (*p* < 0.05). As expected, the strongest CD4^+^ T cell proliferation was obtained in mice immunized with LEX-CD8086 (*p* < 0.01). Furthermore, the levels of IFN-γ and IL-2, which are recognized as indicators of a Th1 response, were measured in the CD4^+^ T cell culture supernatants. We found that upon stimulation with inactivated L1210 cells, the splenic CD4^+^ T cells from mice immunized with LEX-CD80_,_ LEX-CD86, and LEX-CD8086 produced higher levels of IFN-γ (76.13 ± 3.35, 77.12 ± 3.57 and 116.75 ± 8.97 pg/ml, respectively) and IL-2 (64.81 ± 3.76, 69.62 ± 3.42 and 92.15 ± 8.57 pg/ml, respectively) compared to those from mice immunized with LEXnull (45.36 ± 5.37 and 56.60 ± 4.36 pg/ml, *p* < 0.05) (Fig. 3b and c). As expected, CD4^+^ T cells from mice immunized with LEX-CD8086 produced the highest levels of IFN-γ and IL-2 compared to cells from mice in the other groups (*p* < 0.01). Moreover, p388 cells could stimulate neither the proliferation of, nor the Th1 cytokine secretion by CD4^+^ T cells, indicating that the CD4^+^ T cell immune response induced by B7 gene-modified LEXs was antigen-specific.

**Fig. 4:**
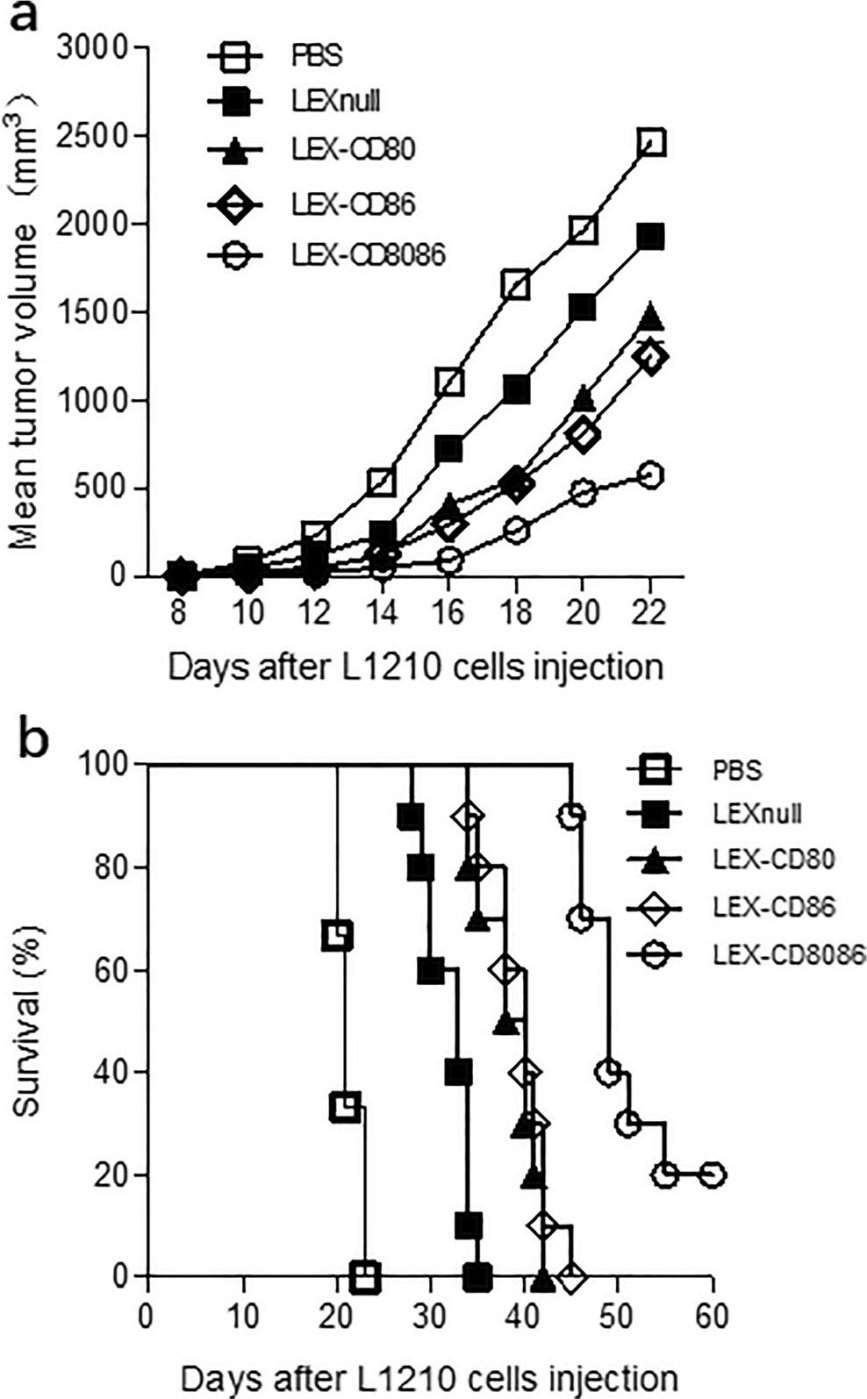
Immunization with B7 gene modified-LEXs induces anti-leukemia immunity. DBA/2 mice were s.c. immunized with 10 μg of each type of exosome formulation or injected with 100 μl PBS on day 0, day7 and day 14. On day 21, mice were s.c. challenged with 5 × 10^5^ L1210 cells. Each group contained 10 mice. Tumor growth (**a**) and survival (**b**) were monitored and recorded after tumor challenge

Next, a cytotoxicity assay was conducted in splenic CD8^+^ T cells that were isolated from the aforementioned immunized mice to verify whether the modified LEXs exhibit a potential activity on CD8^+^ T cell priming and induce antileukemia cytotoxicity. We found that immunization with the modified LEXs could induce a CTL response against L1210 cells compared to the PBS treatment group (Fig. 3d). The cytotoxic activity at an effector to target (E/T) ratio of 50:1 in LEX-CD80 (25.45%)_,_ LEX-CD86 (28.33%), and LEX-CD8086 (42.18%) was stronger than that in the LEXnull group (16.13%, *p* < 0.05). At an E/T ratio of 25:1, the cytotoxic activity against L1210 cells in the modified LEXs groups was still stronger than that in the LEXnull group (*p* < 0.05). As expected, the strongest cytotoxic activity was induced by immunization with LEX-CD8086 (Fig. 4d, compared to that in the LEXnull group, *p* < 0.01). Besides, the CTLs stimulated with all LEX groups did not show any cytotoxic activity against p388 cells, indicating that the antileukemia CTL response induced by these B7 gene-modified LEXs is antigen specific. Taken together, the above results indicate that the modified LEXs could more effectively induce CD4^+^ T cell proliferative responses and Th1 cytokine production, as well as enhance the CTL response in a leukemia antigen-specific manner.

### B7 gene-modified LEXs induce a stronger protective immunity against leukemia cells

To investigate protective antileukemia immunity induced by the modified LEXs in vivo, DBA/2 mice were randomized into 5 groups and s.c. pre-inoculated with PBS, LEXnull or with the three types of B7 gene-modified LEXs, twice with a 7-day interval. The mice were subsequently s.c. challenged with 5 × 10^5^ L1210 cells 7 days after the last vaccination. Tumor growth and overall survival were monitored and recorded. We found that tumor growth in mice immunized with LEX-CD80_,_ LEX-CD86, and LEX-CD8086 was significantly retarded compared to that in the PBS and LEXnull groups (Fig. 4). As expected, the slowest tumor growth was observed in mice immunized with LEX-CD8086, and in some mice the tumors even showed signs of regression (Fig. 4a). Accordingly, the survival of tumor-challenged mice was significantly prolonged by B7 gene-modified LEX vaccination (Fig. 4b). Most mice in the PBS group died approximately 22 days after tumor challenge. The mean survival times (MST) of mice immunized with LEX-CD80 (39 days), LEX-CD86 (40 days), and LEX-CD8086 (49 days) were significantly longer than those of mice immunized with LEXnull (29 days, *p* < 0.05). Two out of ten mice in the LEX-CD8086 group were alive for more than 60 days after tumor challenge, indicating that the immunization with B7 gene-modified LEXs induced strong antileukemia protective immunity, in particular in the LEX-CD8086 group. In summary, our results indicate that modified LEXs overexpressing CD80 and CD86 can induce a more powerful immunity against leukemia cells.

### B7 gene-modified LEXs can efficiently promote the maturation of dendritic cells and facilitate their function

**Fig. 5:**
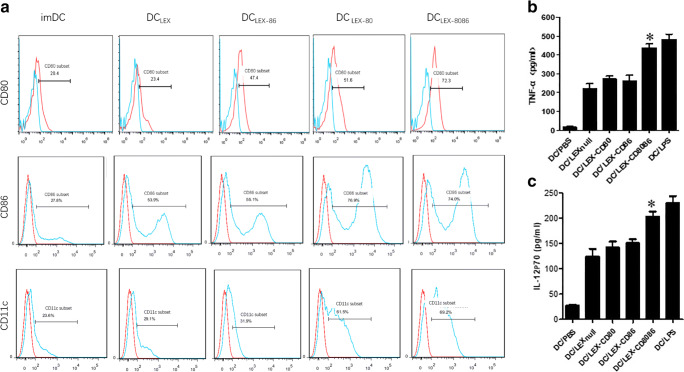
Effect of B7 gene modified-LEXs on immunophenotype and cytokine secretion of DCs. Immature DCs were incubated for 24 h with 10 μg/ml LEXnull, LEX-CD80, LEX-CD86 and LEX-CD8086, respectively. Immature DCs in the absence of stimulation were used as negative controls, while LPS-stimulated DCs were used as positive controls. Expression of CD80, CD86 and CD11c on the surface of DCs was determined by flow cytometry (**a**). The levels of IFN-γ (**b**) and IL-12p70 (**c**) in DC supernatants were measured by ELISA. *p < 0.05 denotes statistically significant difference. Data are representative of three independent experiments and expressed as mean ± SEM

Previously, we found that the immune stimulatory effects of TEXs in vivo depend on the host’s DCs and that DCs treated with TEXs could stimulate efficient anti-tumor cytotoxic T lymphocyte responses, since specific tumor antigens that are carried by exosomes can be transferred to DCs [10, 11, 24]. To investigate the mechanism by which B7 gene-modified LEXs induce a stronger antileukemia immunity in vivo, we next set out to examine the effects of modified LEXs on the immunophenotype and secretion of cytokines in DCs. We found that following incubation with the above LEXs for 24 h, the expression levels of CD80, CD86, and CD11c in DCs were markedly elevated compared to the controls (cells incubated with LEXnull or immature DCs) (Fig. 5). The most significant increases were observed when DCs were stimulated with LEX-CD8086 (Fig. 5a). Furthermore, the secretion of both IL-12 and TNF-α in DCs cocultured with LEX-CD80_,_ LEX-CD86, and LEX-CD8086 was also significantly increased compared to that in immature DCs or in cells incubated with LEXnull. As expected, LEX-CD8086 exerted the most significant stimulation, which was even comparable to the effect of LPS (Fig. 5b, *p* < 0.05). Together, these data suggest that LEXs overexpressing CD80 and CD86 can efficiently promote the phenotypic and functional maturation of dendritic cells, which may be one of the mechanisms through which B7 gene-modified LEXs induce an enhanced antileukemia immune response in vivo.

## Discussion

Although several groups have confirmed the use of TEXs as promising tumor vaccines, the antitumor efficacy of TEXs is still limited [15, 30, 31]. Therefore, improving the efficacy of TEX-based vaccines remains an arduous task. It has been reported that genetically modified tumor cells can be used to develop immunotherapy for leukemia [19, 32]. However, although leukemia cells express leukemia-associated antigens and molecules of the major histocompatibility complexes (MHC) classes I and II, they cannot effectively activate both allogeneic and autologous T cells [33] because they do not sufficiently express costimulatory molecules for T cell activation [19, 22]. The delivery of a “signal 1” by antigen-presenting cells (APCs) to the T cell receptor, in the absence of adequate co-stimulation (“signal 2”), results in T cell anergy and insensitivity to antigen activation. Therefore, upregulation of costimulatory molecules in leukemia cells or APCs is a key issue in the development of leukemia immunotherapy. Several preclinical studies have shown that the introduction of costimulatory molecules in tumor cells enhances their immunogenicity for use as antitumor vaccines [20, 34–36]. Among the costimulatory molecules expressed by “professional” APCs, CD80, CD86, and leukocyte function-associated antigen-3 (LFA-3) play key roles in T cell activation [37, 38].

In this study, we show that the B7 costimulatory molecules CD80 and CD86 exhibit a low expression on L1210 leukemia cells and its exosomes (LEXs). We hypothesized that this low expression could be the main obstacle for LEX-based vaccines to induce efficient anti-leukemia immunity, whereas upregulation of the expression of these molecules in leukemia cells or its LEXs could enhance the antileukemia immunity of LEX-based vaccines. Here, we showed that the expression of costimulatory molecules in leukemia cell-derived exosomes could be upregulated through ectopic expression of costimulatory molecule genes in L1210 leukemia cells. These modified LEXs still express CD9, CD63, and HSP70, indicating that this genetic modification did not affect their biological properties. Therefore, we conclude that it is feasible and convenient to artificially modulate the protein composition of exosomes via genetic modification.

CD4^+^ and CD8^+^ T cells both play unique roles in specific antitumor immunity [39]. Therefore, inducing an effective antigen-specific CD4^+^ T cell and CTL response is the key to LEX-based vaccination. However, TEXs or LEXs alone are not very effective in activating T cells [15, 17, 28], which may be due to insufficient expression of MHC II and costimulatory molecules, since an increase in costimulatory molecules can enhance the immunogenicity of leukemia cells [19, 22]. We next evaluated whether elevation of the expression of costimulatory molecules in LEXs could enhance their antileukemia immunity. We found that the B7 gene-modified LEXs overexpressing CD86 and CD80 efficiently induced CD4^+^ T cell proliferation and promoted secretion of Th1 cytokines in an antigen-specific manner. Increased Th1 cytokines can potentially induce better CTL responses. Concordantly, we found that leukemia cell antigen-specific CTL activity was most significantly enhanced in mice immunized with the modified LEXs. It has been reported that, despite DC-mediated CTL activation, exosomes are taken up by CD8^+^ T cells and directly induce antigen-specific cytotoxic activity of CD8^+^ T cells via exosomal MHC molecules [40–42]. Therefore, the enhanced CTL response observed here may be directly mediated by the B7 gene-modified LEXs through overexpression of costimulatory molecules.

To verify if the modified LEXs overexpressing CD86 and CD80 exerted a positive effect on immune cells, we investigated the antileukemia immunity induced by these LEXs in animal models. We found that the modified LEXs showed superiority in inhibiting tumor growth as well as in prolonging survival. These data indicate that costimulatory molecule gene-modified LEXs can more efficiently induce anti-leukemia immunity compared to non-modified LEXs. Among these modified LEXs, the most efficient stimulatory effect on immune cells, the induction of a CTL response and antileukemia protective immunity in the animal model was exerted by LEX-CD8086, indicating that an enhanced immunogenicity of LEXs was achieved via infection with vectors encoding multiple costimulatory molecules.

Previous studies have suggested that APCs can capture lipoproteins or tumor cell-associated antigens (TAA) and thus, that the expression of costimulatory molecules and cytokine secretion would be significantly enhanced [43, 44]. CD80 and CD86 are two major costimulatory molecules that play significant roles in T cell priming for IL-2 and IFN-γ production. Hence, it is reasonable to speculate that overexpression of CD80 and CD86 may boost the effects of LEXs on the immune system. The majority of mice immunized with B7 gene modified LEXs did, however, not show long-term survival during our observation period, suggesting that the overexpression of costimulatory molecules alone in LEX-based vaccines may still not be sufficient to inhibit tumor progression. Therefore, further optimization of LEX-based vaccines is still required.

We and others have reported that TEXs are predominantly internalized by DCs and directly influence their maturation and function [15, 29, 45]. DCs are major APCs and are responsible for activating both CD4^+^ and CD8^+^ T cells against tumors [46]. Previously, we also suggested that the immune stimulatory effects of TEXs are dependent on the host’s DCs [15, 47]. Therefore, it remained to be explored whether overexpression of costimulatory molecules in LEXs can enhance DC function. We found that similar to other TEXs, B7 gene modified-LEXs can be taken up by DCs (data not shown). Therefore, a LEX-carried antigen profile can be transferred to DCs and may induce antigen-specific antileukemia immunity. Furthermore, we found that coculture with these B7 gene modified-LEXs could markedly elevate the expression of surface costimulatory molecules and promote the secretion of cytokines by DCs, suggesting that the expression of costimulatory molecules in LEXs can enhance the maturation and function of DCs. These results also suggest that overexpression of costimulatory molecules in LEXs may reverse the immune suppressive effect caused by LEX-induced obstruction of DC maturation and activation. Thus, overexpression of costimulatory molecules in LEXs may be effective for boosting DC function and antileukemia immunity.

CD80 and CD86 are double-edged swords, because CD28 and cytotoxic T lymphocyte-associated protein 4 (CTLA-4) simultaneously share them. CTLA-4 signaling is crucial for T cell activation, can decrease the activity of helper T cells (Th1) and enhance the immunosuppressive activity of regulatory T cells when combined with CD80 and CD86 [48]. Other immunosuppressive molecules, such as Fas ligand and IL-10 can interfere with the immunogenicity of TEX-based vaccines [48]. Recently, we found that the anti-leukemia immunity of LEXs can be enhanced via silencing or downregulation of TGF-β1 expression [31]. Therefore, our next step is to combine the reversal of inhibitory effects of immunosuppressive molecules through silencing or downregulation of TGF-β1 with the overexpression of costimulatory molecules in LEX-based vaccines to amplify TCR signaling and activate T cell proliferation. Overall, our study provides a novel and promising basis for optimizing LEX-based vaccines.

## Abbreviations

DC: Dendritic cell
TEX: Tumor-derived exosome
LEX: Leukemia-derived exosome
HSP: Heat shock protein
IL-2: Interleukin 2
TGF-β1: Transformation growth factor-1
TNF-α: Tumor necrosis factor-α
IFN-γ: Interferon-γ
CTL: Cytotoxic T lymphocyte
APC: Antigen-presenting cell
CTLA-4: Cytotoxic T lymphocyte-associated protein 4
